# The Importance of Cellular Metabolic Pathways in Pathogenesis and Selective Treatments of Hematological Malignancies

**DOI:** 10.3389/fonc.2021.767026

**Published:** 2021-11-10

**Authors:** Mojdeh Soltani, Yue Zhao, Zhijia Xia, Mazdak Ganjalikhani Hakemi, Alexandr V. Bazhin

**Affiliations:** ^1^ Department of Immunology, Faculty of Medicine, Isfahan University of Medical Sciences, Isfahan, Iran; ^2^ Department of General, Visceral, and Transplant Surgery, Ludwig-Maximilians-University Munich, Munich, Germany; ^3^ German Cancer Consortium (DKTK), Partner Site Munich, Munich, Germany

**Keywords:** acute lymphocytic leukemia, acute myeloid leukemia, chronic lymphocytic leukemia, chronic myeloid leukemia, cellular metabolism, immunometabolism

## Abstract

Despite recent advancements in the treatment of hematologic malignancies and the emergence of newer and more sophisticated therapeutic approaches such as immunotherapy, long-term overall survival remains unsatisfactory. Metabolic alteration, as an important hallmark of cancer cells, not only contributes to the malignant transformation of cells, but also promotes tumor progression and metastasis. As an immune-escape mechanism, the metabolic adaptation of the bone marrow microenvironment and leukemic cells is a major player in the suppression of anti-leukemia immune responses. Therefore, metabolic rewiring in leukemia would provide promising opportunities for newer therapeutic interventions. Several therapeutic agents which affect essential bioenergetic pathways in cancer cells including glycolysis, β-oxidation of fatty acids and Krebs cycle, or anabolic pathways such as lipid biosynthesis and pentose phosphate pathway, are being tested in various types of cancers. So far, numerous preclinical or clinical trial studies using such metabolic agents alone or in combination with other remedies such as immunotherapy are in progress and have demonstrated promising outcomes. In this review, we aim to argue the importance of metabolic alterations and bioenergetic pathways in different types of leukemia and their vital roles in disease development. Designing treatments based on targeting leukemic cells vulnerabilities, particularly in nonresponsive leukemia patients, should be warranted.

## Introduction

Hematologic malignancies are heterogeneous disorders caused by the carcinogenic transformation of hematopoietic cells. Leukemias are the most common set of these malignancies characterized by rapid and aggressive production of abnormal white blood cells (WBCs) that are not functional and efficient immune cells. This state finally eventuates to impairment of the bone marrow ability to produce sufficient and beneficial blood cells like platelets, red blood cells (RBCs), and normal WBCs because of space occupation (1–3). According to the American Cancer Society, about 60,530 new cases and approximately 23,100 deaths of leukemia were estimated in the United States in 2020 (4). Despite significant improvements in its treatment and the advent of newer and more efficient therapies such as immunotherapeutic approaches, long-term survival remains unsatisfactory and more investigation in this field is still a requisite. There are several categories of leukemia based on various criteria, but according to the most well-known one, leukemia can be divided into lymphocytic and myelogenous (or myeloid) groups (1).

Metabolic alteration is a significant hallmark of cancer cells which enables them to fulfill their requirements. Actually, metabolic adaptations not only contribute to cancer cells transformation, but also promote tumor progression (5, 6). It seems that metabolic rewiring is also a critical implement for leukemic cells so that metabolic properties of these cells can remarkably influence the disease progress and effective response to treatments. Indeed, both cell-intrinsic and extrinsic factors including oncogenic mutations and some aspects of the bone marrow microenvironment (BMM) significantly affect leukemic cells’ metabolism. The bone marrow and thymus, where the leukemia and lymphoma cells expands, are hypoxic areas with specific metabolic condition (7–9). Metabolic characteristics of leukemia cells, particularly leukemic stem cells (LSCs) that are usually different from normal hematopoietic stem cells (HSCs), actively contribute to the initiation and maintenance of malignancy. Furthermore, metabolic adaptations in the BMM and leukemic cells have a critical role in the suppression and eradication of anti-leukemia immune responses (10, 11). Regarding these issues, metabolic vulnerabilities in leukemia provide promising opportunities for therapeutic intervention. In this review, we try to discuss the importance of metabolic regulations and bioenergetic pathways in leukemia pathogenesis and their vital roles in disease development. In addition, designing of treatment based on targeting leukemic cells vulnerabilities, particularly in nonresponsive leukemia patients, is considered.

## Classification of Leukemia and Common Therapies

According to several references, leukemias can be classified as acute and chronic as well as myelocytic and lymphocytic based on the degree of cell differentiation and the predominant type of cells involved (12). Acute myeloid leukemia (AML) which accounts for approximately 33% of leukemia new cases in the US, is the most frequent and worst prognostic type. According to the French–American–British classification, AML is divided into eight subtypes (M0–M7) (13). It occurs because of some genetic abnormalities in myeloblasts, which leads to bone marrow function failure eventually. A combination of cytarabine plus anthracycline cytotoxic chemotherapy has been the standard therapy of AML since the past three decades (14, 15). Despite advances in AML therapy, its prognosis is still poor, especially for older leukemia patients. Therefore, along with chemotherapeutic strategies, some immunotherapeutic approaches such as immune checkpoint blockade and multi-specific T cell-engaging antibodies, are under investigation (16–18). Chronic myeloid leukemia (CML) is a clonal myeloproliferative disease that accounts for about 15% of adult leukemia cases (13). It is characterized by aggressive proliferation of dysfunctional myeloid-derived cells and is classified into three stages including the chronic phase, accelerated phase, and blastic crisis (19). The overexpression of BCR-ABL1 fusion oncogene, which is resulted from a reciprocal translocation between chromosome 9 and 22 (Philadelphia chromosome) in HSCs, eventually gives rise to a constitutively active non-receptor tyrosine kinase activity. This incident is considered as the main origin point of the disease, especially in the chronic phase (20, 21). Hence, tyrosine kinase inhibitors (TKIs) which are classified into three generations, are the main standard treatment and have remarkably improved the outcomes of CML patients in the chronic phase. Nevertheless, almost 50% of CML patients suffer a relapse of disease post-TKI therapy due to the presence and drug-resistance of TKI-resistant CML stem cells (22, 23).

Acute lymphoblastic leukemia (ALL) is known as the most common leukemia in pediatrics with an approximate prevalence of 80% childhood leukemias, however, its adult‐onset form mostly involves males age >70 years (24, 25). ALL arises from the transformation of hematopoietic B cell or T cell progenitors with chromosomal abnormalities or genetic alterations. Not only genetic but also environmental predisposing factors, like childhood infections, will contribute to the initiation and progression of ALL. Although the 5-year overall survival (OS) of pediatric ALL patients has increased from 31% in 1975 to nearly 70% in 2009 and even to 90% in recent years, only 25% of patients older than 50 years old live longer than 5 years after diagnosis. That shows urgent needs for further improvements for its treatment particularly the adult form (25). Accordingly, frontline treatment of ALL includes four phases during 2–3 years: induction, consolidation, intensification, and long-term maintenance. Asparaginase therapy, as a frontline approach to cytoreduction, is administered in all cases of pediatric and even most cases of adult ALL and this issue declare the importance of cellular metabolism in its pathogenesis and treatment. Asparaginase is considered a critical component used for the treatment of childhood ALL and can give rise to complete remission in 40%–60% of patients, while the 5-year OS in older patients (>60 years old) is still poor, even less than 20% (25–27). The last type of leukemia is chronic lymphocytic leukemia (CLL) that is considered as the most common adult leukemia accounting for approximately 40% of all cases in adults. It results from an overproduction of abnormal B cell lymphocytes in the bone marrow that accumulate in the bloodstream and homing tissues. Even though it is known as slow-progressing leukemia, CLL is somehow incurable, and drug resistance or relapse is often presented in CLL patients (28).

## The General Importance of Metabolic Pathway in Cancer Therapy

To fulfill the bioenergetic and biosynthetic needs, some alterations occur in the energy metabolism of cancer cells. These metabolic shifts are mainly triggered by various mechanisms which instigate signaling pathways and eventually manipulate the expression of some metabolism-related genes (29). Cancer cells generally have three metabolic hallmarks including increased glycolysis, glutaminolysis, and lipogenic pathways (30). It has been reported that the phosphoinositol 3-kinase (PI3K)-AKT signaling pathway, which activates mammalian target of rapamycin (mTOR), hypoxia-inducible factor 1 (HIF1) and other transcription factors like c-Myc, p53, or Oct1, has a critical role in these metabolic adaptations (29, 30). Cellular metabolism is mainly controlled by mTORC master regulator that affects the translation and transcription of metabolic genes, such as peroxisome proliferator activated receptor γ coactivator-1 α (PGC-1α), sterol regulatory element-binding protein 1/2 (SREBP1/2), and hypoxia inducible factor-1 α (HIF-1α). This regulator complex not only promotes glycolytic activity that contributes to cell growth but also, interestingly, can regulate mitochondrial metabolism. Thus, representing an important regulator in mitochondrial function (31–33). Fortunately, these metabolic rewirings of cancer cells have provided promising drug targets during the past decades. Several therapeutic agents which affect either requisite bioenergetic pathways or anabolic pathways in leukemia cells are being applied in various types of cancers. Numerous preclinical studies or clinical trials using these metabolic agents alone or in combination with other remedies such as immunotherapy are in progress and have demonstrated promising outcomes (5).

## Metabolic Differences of Normal Hematopoietic and Leukemic Cells

Metabolic differences between normal hematopoietic and leukemic cells can provide new therapeutic windows to treat leukemia patients. HSC self-renewal and lineage differentiation potency are not only influenced by cytokines and transcription factors but also regulated by cell metabolism (7, 10). In the dormant state, low levels of ROS and ATP production due to the presence of anaerobic glycolysis were observed in HSCs. However, when switching to an active state, HSCs usually utilize oxidative metabolism through elevated levels of oxidative phosphorylation (OXPHOS) and fatty acids oxidation (FAO) due to a higher energy demand (34, 35). Actually, because of the low oxygen microenvironment of bone marrow, HIF-1s are stabilized and promote a glycolytic phenotype in HSCs mostly through the stimulation of the pyruvate dehydrogenase kinase (PDK) 2 and 4 as an active preventer of the TCA cycle (8, 36). Additionally, asymmetric division of HSCs which is requisite for the production of eternal daughter cells and committed progenitors is considerably dependent on PML-peroxisome proliferator-activated receptor delta (PPARδ)-FAO (37). According to previous studies, it seems that although LSCs may have some similar metabolic properties to normal HSCs, there are also some differences that exist. Lagadinou et al. have demonstrated that LSCs have a higher expression of BCL-2 and thus, contain lower ROS levels than HSCs (38). Some other investigations have demonstrated metabolic differences between leukemic cells and their normal counterparts. Zhong et al. have reported that T-ALL leukemic cells show increased OXPHOS in comparison to normal HSCs (39). Increased free fatty acids utilization and energy production by OXPHOS has been detected in CLL cells unlike normal B-lymphocytes (40). Interestingly, AML cells have more mitochondrial mass but lower respiratory chain complex activities and spare respiration in comparison to normal CD34+ cells (41, 42). On the other hand, a high level of glucose consumption is considered a conserved feature of LSCs in most hematological malignancies, regardless of genetic defects or types of them. Upregulation of glycolysis has been indicated in several kinds of leukemia including AML, ALL, CLL, and multiple myeloma (43–46). Moreover, it has been indicated that increased glycolysis correlates with drug resistance in both leukemia patients and cell lines (47). Oncogenic drivers in human leukemia including MYC and RAS are major operative factors in the metabolic rewiring of leukemia cells. For instance, MYC overexpression is considered a significant hallmark of B-cell and T-cell ALL and also Burkitt’s lymphoma, which can actively promote some metabolic pathways such as glycolysis, mitochondrial biogenesis, glutaminolysis, lipid, and nucleotide biosynthesis (48–52).

## Importance of Cellular Metabolic Pathways in Myeloid Disorders

### Glucose Related Metabolic Pathways

**Figure 1:**
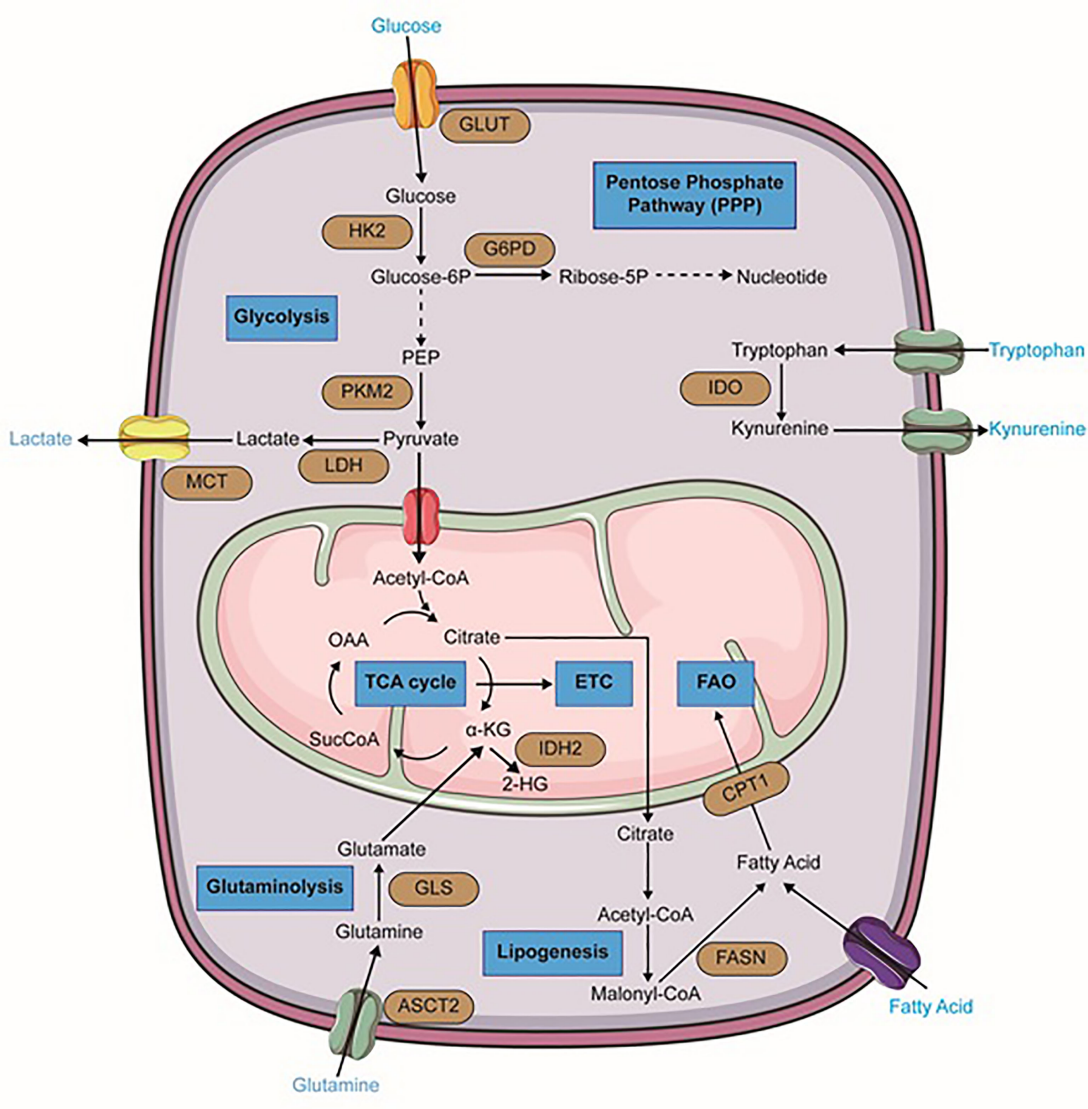
Metabolic alterations in leukemia cells. Important reactions of the metabolic pathway in leukemia cells are summarized in the schematic, including glycolysis, glutaminolysis, lipogenesis, TCA cycle, fatty acid oxidation (FAO), pentose phosphate pathway (PPP) (blue). Dominant nutrient transporters and metabolic enzymes are emphasized (brown). GLUT, glucose transporter; HK2, hexokinase-2; PKM2, pyruvate kinase isozymes M2; LDH, lactate dehydrogenase; MCT, monocarboxylate transporters; G6PD, glucose-6-phosphate dehydrogenase; ASCT2, glutamine transporter encoded by Solute Carrier Family 1 Member 5 gene; GLS, glutaminase; FASN, fatty acid synthase; CPT1, carnitine palmitoyltransferase I; IDH2, isocitrate dehydrogenase 2; IDO, Indoleamine-pyrrole 2,3-dioxygenase; α-KG, alpha-ketoglutarate; SucCoA, Succinyl-CoA; OAA, oxaloacetate. The figure was produced with the assistance of Servier Medical Art (https://smart.servier.com).

Regarding the critical role of glycolysis pathway in the growth and proliferation of cancer cells, myeloid leukemia cells also upregulate this metabolic pathway. Numerous genetic studies in mouse models have implicated the importance of glycolysis and high expression of glycolysis-associated enzymes in the initiation and maintenance of myeloid malignancies. They have proved that deletion of glycolytic enzymes such as pyruvate kinase (PKM2) or lactate dehydrogenase A (LDHA) can considerably extenuate the initiation and maintenance of murine CML and AML (53). Raised glycolytic activity not only gives rise to a decreased level of autophagy leading to more aggressive leukemia, but also is associated with drug resistance (54, 55). In AML cells, higher mTORC1 activity and FLT3-ITD-mediated signaling contribute to the continuance of glycolysis and leukemic cells survival and therefore, render therapeutic sensitivity to glycolytic inhibition (**Figure 1**) (56, 57). 2-Deoxyglucose, as an inhibitor of the glycolysis pathway, has shown beneficial effects in AML treatment (47, 58). Moreover, leukemic cells are significantly dependent on the pentose phosphate pathway (PPP) which provides ribonucleotides for nucleic acid synthesis and NADPH for lipid synthesis. Redox homeostasis and glucose-6-phosphate dehydrogenase (G6PD) overexpression are the key regulators of this pathway and correlate with an adverse prognosis in AML (**Figure 1**). Therefore, inhibition of G6PD activity can be a promising therapeutic target in AML and its beneficial effects have been demonstrated in FLT3-ITD-mutated AML (56, 59, 60). Additionally, synergic effects of G6PD and 6-phosphogluconate dehydrogenase (6PGD) inhibition with cytarabine to produce anti-leukemic activities in AML have been proven (42). BCR‐ABL fusion oncogene in CML also increases glucose transport and renders cells more glycolytic compared with normal counterparts (61). It has been demonstrated that continuous exposure of K562, a CML cell line, with imatinib which decreases glucose uptake and inactivates glycolytic enzymes, can lead to induction of autophagy in CML cells by mechanisms involving activation of AMPK and suppression of S6K1 (62).

OXPHOS and mitochondrial metabolism also have a significant role in myeloid leukemia. According to Krtić et al, oxygen consumption rate and mitochondrial mass are increased in AML cells compared with normal hematopoietic progenitors (63). Furthermore, amplification of mitochondrial DNA (mtDNA) levels in AML has been indicated in various studies (64, 65). Noteworthily, different AML cell lines exhibit a different preference on glycolysis or mitochondrial OXPHOS for energy demand. For instance, NB4 was proved to be a “glycolytic” cell line while THP-1 was identified to be dependent on OXPHOS, even though they are all AML cell lines (66). Further mechanism investigation revealed that AMPK is responsible for the bioenergetic production difference, highlighting that the oncogene background of leukemia cells, at least partly, determines their metabolism preference. Furthermore, leukemia continuously reprograms to adapt to environmental pressures and alteration of growth conditions, resulting in the ratio between glycolysis and OXPHOS are continuously changing (67). Due to higher mitochondrial biogenesis and higher dependency on mitochondrial oxidation in leukemia myeloblasts, it has been indicated that metformin suppresses leukemia cell survival, proliferation, and clonogenic activity in both AML and CML (68, 69). The compound CPI-613, as an inhibitor of pyruvate dehydrogenase (PDH) and α-KG dehydrogenase (KDH), has clinical benefits through decreasing oxygen consumption rate in AML cells (70). An unexpected high remission rate has been observed in relapsed and refractory AML patients after co-administration of CPI-613 with high-dose Ara-C (71). Several OXPHOS or ETC complexes inhibitors including Tigecycline, Metformin, 2’3’-Dideoxycytidine, IACS-010759, and A2-32-01 have revealed clinical benefits in AML and CML (63, 65, 72, 73). Metformin, as a widely used anti-diabetic drug, has interestingly exhibited anti-leukemic activities in some leukemias such as AML. However, regarding its pharmacokinetics and maximum efficient dose, it is usually administered in association with other remedies but not alone in clinical settings. Regarding that AML cells are strongly dependent on mitochondrial metabolism, metformin is administered in doses which could inhibit ETC complex I (69, 74). It has been proven that in order to eliminate disease persistence in CML patients, a combination of tigecycline and imatinib can serve as a suitable clinical approach (75).

One of the most important and well-known metabolism-related hallmarks of AML is a mutation of isocitrate dehydrogenases. These are crucial enzymes of the TCA cycle. In their wild-type form, these isoforms oxidatively decarboxylate isocitrate to 2-KG and generate NADPH. However, their mutant forms oxidize NADPH and produce the oncometabolite 2-hydroxyglutarate (2-HG) from α-KG (**Figure 1**) (76–78). This alteration has been found approximately in 20% of AML patients and has been proven to be a promising therapeutic point. In 2018, ivosidenib (formerly known as AG120), an allosteric IDH1^R132^ inhibitor, was firstly approved by the FDA for patients with relapsed or refractory IDH1-mutated AML. Additionally, it is considered as front-line therapy for newly diagnosed patients who are 75 years or older and also some patients who are ineligible to receive intensive chemotherapy (79, 80). Enasidenib (formerly known as AG-221) that has gained FDA approval for IDH2 mutant AML patients, is a small molecule that selectively inhibits mIDH2 (IDH2^R140Q^ and IDH2^R172K^) *via* binding to its allosteric site and prevents the conformation change requiring for the catalytic action and the R-2-hydroxyglutarate (R2HG) production. Its clinical benefits in AML treatment have been demonstrated in both in-vitro studies and clinical trials (80–82). Furthermore, Vorasidenib (AG-881) is under investigation in phase I for the treatment of AML patients with a mutation in IDH1 and/or IDH2 (83).

### Amino Acids and Protein Related Metabolic Pathways

Amino acids not only serve as essential building blocks of protein biosynthesis but also are considered as a significant source of carbon and nitrogen on nucleotide synthesis and therefore they contribute to cancer cells’ growth and proliferation. According to numerous studies, it seems that three amino acids glutamine, arginine, and tryptophan have prominent roles in hematological malignancies particularly in myeloid leukemia. The importance of branched−chain amino acids (BCAAs) metabolism in myeloid leukemia has also been studied. Accordingly, in several leukemic cell lines, glutamine depletion can induce significant apoptosis (84–86). AML primary cells have indicated a higher level of glutamine dependence than normal CD34+ cells because this amino acid controls OXPHOS in these cells (87). Small molecule inhibitors such as BPTES and CB-839 that are glutaminase (GLS) inhibitors, can lead to cell proliferation arrest and apoptosis in AML and BCR–ABL-positive CML cells (88, 89). CB-839 can also inhibit glutathione production and cause accumulation of mitochondrial ROS and eventually apoptotic cell death in AML (90). Recent research showed that high-risk MDS patients can benefit from the combination of CB-839 and 5-azacitidine due to the rapid reduction of BM blasts (91). Moreover, GLS inhibition and BCL-2 inhibitor Venetoclax have displayed synergic effects in AML (**Figure 1**) (86).

Various studies also described a strong dependence of myeloid leukemia cells on arginine. Arginine is a semi-essential amino acid that is involved in many biochemical processes and can be considered as a potential therapeutic cancer target (92–94). Because of defects in the arginine-recycling pathway enzymes like argininosuccinate synthase and ornithine transcarbamylase, AML blast cells are mostly dependent on arginine uptake by the CAT-1 and CAT-2B arginine transporters. In addition, the plasma arginine levels are significantly higher in AML patients compared with healthy counterparts. Hence, BCT-100, a PEGylated human recombinant arginase that can deplete extracellular arginine, has resulted in proliferation arrest, apoptosis, and decreased AML engraftment *in vivo*. Generally, the cultivation of AML blasts or AML cell lines in an arginine-free media can lead to their apoptosis (94).

Tryptophan metabolism and its conversion to kynurenine, have an important role in a variety of malignant tissues, such as ovarian cancer, melanoma, and head and neck cancer (95). Increased kynurenine/tryptophan ratio in AML patient sera which negatively correlates with their OS, also indicates its importance in myeloid leukemia (96). In fact, in more than 50% of the cases tested at diagnosis, AML blasts constitutively express IDO (Indoleamine-2,3-dioxygenase) and so can promote the formation of regulatory T cells (Tregs) (97).

leucine, isoleucine, and valine are known as BCAAs synthesized by the BCAA transaminases 1 (BCAT1) and 2 (BCAT2) in a reversible transamination reaction. A quantitative expression proteomics analysis detected significant BCAT1 overexpressed in AML LSCs but not in the non-LSC bulk population. A high level of BCAT1 is involved in AML biology (98). Furthermore, Hattori et al. have indicated highly elevated BCAA levels in CML-initiating cells isolated from a BC-CML mouse model using amino acid-specific fluorescent markers. They also have shown that BCAT1 inhibition impairs CML blast crisis propagation *in vitro* and *in vivo* (99).

### Lipid Related Metabolic Pathways

In a general perspective, lipids are considered as central players in cancer biology due to their critical roles in cell growth and proliferation. Therefore, extensive rewiring of lipid metabolism, which is driven by both oncogenic and tumor microenvironmental cues, is a significant hallmark of cancer cells. Indeed, these metabolic reprogramming and specific lipid profiles of cancer cells are so important that can be regarded as disease biomarkers, with diagnostic, prognostic, and predictive values. Lipids and their metabolism are emerging as promising tools and targets for anti-cancer therapies and numerous natural or synthetic compounds targeting lipid metabolism have been discovered with curative effects against cancer (100, 101). Interestingly, bone marrow-resident LSCs express higher fatty acid transporter CD36 than normal HSCs and hence, they can induce lipolysis in BM adipocytes to fuel FAO in leukemic cells. It seems to be that BM-resident adipocytes remarkably impair the anti-leukemia effect of various chemotherapeutic agents and obese mice compared to normal-weight counterparts show higher rates of relapse after chemotherapy (102). It has been proven that cell growth and survival of AML monocytes are strongly supported by BM adipocytes, which promote their fatty acid β-oxidation (103). Firstly, a study in 2010 showed that pharmacological inhibition of the carnitine palmitoyltransferase 1 (CPT1) which catalyzes one of the rate-limiting steps in FAO, enhanced AML sensitivity to apoptosis (**Figure 1**) (104). Moreover, other pharmacologic agents that inhibit fatty acid oxidation, such as Etomoxir or Ranolazine, restrain AML cell proliferation and sensitize human leukemia blasts to apoptosis (104). Even some more common lipid targeting drugs like statins are also clinically useful in AML treatment in combination with chemotherapy. Some studies have demonstrated that statin treatment can enhance complete remission rates in favorable-risk AML groups, but had a less clear role in high-risk AML (105, 106).

Lipids are not only a source of energy to produce ATP, NAD(P)H, and building blocks for cell membrane, but also are essential signaling molecules. This group of lipids including prostaglandins, leukotrienes, and eicosanoids has a critical role in several processes like inflammation, and carcinogenesis (107). Chen and colleagues have demonstrated that mRNAs for both Alox5 and Alox15 (arachidonic acid lipoxygenase enzymes) are highly expressed in CML stem cells and their overexpression is vital for their survival. Additionally, they showed that *in vivo* administration of PD146176 (an Alox15 inhibitor) and/or zileuton (an Alox5 inhibitor) can diminish the relapse of CML disease in WT CML model mice (108, 109). Another study has indicated that increased expression of the enzyme Gdpd3 (lysophospholipase D), which activates lysophospholipid metabolism, can eventually lead to stem cell quiescence and TKI resistance in CML. These observations suggest that targeting Gdpd3 or other enzymes that are involved in lysophospholipid metabolism can contribute in reducing CML disease relapse (110).

## General Alterations of Metabolites in Lymphoid Leukemia

### Glucose Related Metabolic Pathways

Although different hematological malignancies harbor individual genetical alterations and locate in a diverse microenvironment, ALL shows a conserved symptom of high consumption of glucose as myeloid leukemia does (111). B precursor ALL lymphoblasts exhibit a preferential upregulation of genes related to glycolysis compared to the TCA cycle, indicating the dominant position of aerobic glycolysis in glucose metabolism of ALL (111). However, in contrast to ALL, CLL shows a relatively low uptake of 2-Deoxy-2-[^18^F] fluoroglucose (FDG) and low sensitivity of positron emission tomography-FDG, demonstrating that the glycolytic pathway might not be a critical role in CLL cells’ metabolism (112, 113). As the glucose transporter, GLUT promotes the uptake of glucose extracellularly in ALL cells, and after deletion, a decrease in proliferation and a limited degree of apoptosis was observed in B cell ALL lymphoblasts harboring the BCR-ABL1 tyrosine kinase fusion oncogene (114). Moreover, inhibition of rate-limiting enzymes that catalyze the metabolic intermediates during glycolysis, such as HK2, PKM2, and LDHA, induces apoptosis, blocks proliferation, and even attenuates drug resistance in ALL cells. Therefore, opens a potential therapeutic window for ALL treatment by targeting crucial enzymes in the glycolysis pathway (114, 115). Numerous oncogenic pathways, such as MYC, PI3K/Akt, AMPK, and Notch, have been reported to participate in the glycolysis procedure in lymphoid leukemia, upregulating the expression of glucose transporters and glycolytic enzymes or balancing glycolysis and mitochondrial metabolism (116–118).

The PPP, which branches from glycolysis, produces NADPH and ribose-5-phosphate after an oxidative phase and plays an unreplaceable role in lipids generation and redox homeostasis in lymphoid leukemia (119). G6PD, the first enzyme in the PPP pathway, can serve as a prognostic biomarker of ALL (120). Furthermore, in B cell ALL, the serine/threonine-protein phosphatase 2A (PP2A) switches glucose carbon utilization from glycolysis to the PPP to cope with oxidative stress, implicating the possibility to remove the gatekeeper function of PP2A in the PPP by inhibiting this molecule in ALL (121).

Despite the roles of aerobic glycolysis as a predominant pathway for providing a source of energy and anabolic precursors for tumorigenesis in most hematopoietic malignancies, some subsets of leukemia, like CLL, satisfy from increasing energy demands during growth by upregulating mitochondrial OXPHOS activity. For instance, an increase in the number of mitochondria, the total mitochondrial mass, and mitochondrial biogenesis was evidenced in CLL cells, in comparison to normal B lymphocytes (122). Elevated mitochondrial oxidative activity generates a proper high level of ROS, contributing to tumor progression by mediating the tumor microenvironment and protecting cells from apoptosis. When compared with normal B cells, the level of mitochondria-derived ROS in CLL cells is higher due to a more active mitochondrial activity, thereby promoting the progression of leukemia (40, 122). Metformin, an inhibitor of mitochondrial complex I, has been proved to suppress cell cycle progression and cell proliferation *via* disruption of mitochondrial OXPHOS in CLL and ALL (123, 124). Furthermore, a synergetic antileukemia effect has been observed when combining metformin with daunorubicin or vincristine, which are extensively used to treat leukemia clinically. This has revealed that, revealing that targeting mitochondrial OXPHOS could be a potent adjuvant therapy strategy for lymphoid leukemia treatment (125, 126).

### Amino Acids and Protein Related Metabolic Pathways

Glutamine provides a rich resource of carbon and nitrogen, which can be used to produce amino acids, nucleotides, and fuel mitochondrial activity *via* offering α-KG. Thus, many cancer cells become addicted to glutamine and rely heavily on glutaminolysis for rapid growth. Likewise, ALL cells show an active utilization of glutamine for leukemogenesis, while suppression of glutaminolysis enhances the antileukemic effects in mice harboring T cell ALL (127). Genetic or pharmacological targeting ASCT2 molecule, a transporter of neutral amino acids, especially glutamine, decreases leukemia initiation and maintenance driven by the oncogene MLL-AF9 or Pten deficiency (128). Furthermore, glutaminase (GLS), An enzyme that catalyzes glutamine into glutamate, has proved to be a potential therapeutic target in the treatment of ALL (129, 130).

Asparagine synthetase (ASNA) catalyzes the conversion from aspartate to asparagine in most tissues. However, due to the low expression of ASNA, ALL cells are auxotrophic for asparagine and highly sensitive to asparaginase treatment, which degrades extracellular asparagine systematically and blocks the cell growth of ALL cells (131, 132). Taking advantage of this unusual metabolic dependency on an exogenous supply of asparagine in ALL cells, asparaginase has become one of the standard therapeutic regimens in ALL treatment. Asparaginase already exhibited a synergetic and profound antileukemia effect when combining with chemotherapies for ALL treatment in clinical practice (133, 134). Intriguingly, asparaginase diminishes not only asparagine but also glutamine in the intracellular pool (130). Moreover, asparaginase reduces glucose uptake and glycolysis and hampers pyrimidine synthesis in ALL cells (135). Altogether, asparaginase can fulfill its antileukemia function in a comprehensive method.

Arginine is a semi-essential amino acid, which is critical for the synthesis of protein, nitric oxide, and other amino acids. Arginine can be synthesized from citrulline intracellularly while taking up exogenous arginine is also necessary to meet the cellular demands in leukemias. Increasing studies have shown the potential therapeutic benefit of pegylated-human-arginase I (peg-Arg I) in ALL treatment. By depleting the existence of arginine and starving ALL cells of it, peg-Arg I induces a decreased progression of the cell cycle *via* impairing the expression of cyclin D3 (136). Further study suggested the central role of phosphorylation of the eukaryotic-translation-initiation factor 2 alpha signaling in the antileukemia effects induced by peg-Arg I (137). Interestingly, although endogenous or genetic upregulation of argininosuccinate synthase or ornithine transcarbamylase rescues ALL survival from a moderately low arginine condition, these enzymes cannot prevent the cytotoxic effects of peg-Arg I towards ALL (138).

### Lipid Related Metabolic Pathways

Lipids serve as fundamental building blocks for the construction of membranes. Cancer cells satisfy the high demands of lipids for the biosynthesis of membrane structure *via* synthesizing fatty acids themselves, rather than absorbing lipids from the blood. Fatty acid synthase (FASN), a large enzyme with multiple catalytic domains, carboxylates malonyl-CoA into fatty acid chains. An elevated level of FASN was evidenced in drug-resistant ALL and correlated with a poor prognosis, while inhibition of FASN increases apoptosis rates and overcomes dexamethasone resistance (139). Moreover, not only the suppression of FASN inhibits ALL cells growth but also produces a remarkable impairment of PD-L1 expression, indicating the potential role of FASN inhibitors in antileukemia immunotherapy (140).

FAO in the mitochondria degrades fatty acids and generates acetyl-CoA for OXPHOS and energy production in cancer cells. The transportation of fatty acids into mitochondria for FAO requires the assistance of CPT, an enzyme located in the outer membrane of mitochondria. CLL cells exhibit elevated levels of CPT1 and CPT2, while inhibition of CPT induces compromised FAO activity due to ineffective transportation of fatty acids into mitochondria, subsequently leading to massive CLL cell death (141).

Lipoprotein lipase (LPL), a water-soluble enzyme that hydrolyzes triglycerides in lipoproteins, has found to be abnormally expressed in CLL cells (142). Moreover, the elevated expression of LPL is associated with a poor prognosis and an aggressive disease stage of CLL (142, 143). LPL displays its oncogenic function *via* storing lipids in cytoplasmic vacuoles and fueling fatty acids for energy production, reprograming CLL cells to utilize fatty acids as the major energy resource (144). Furthermore, this unique metabolic feature of CLL cells is driven by STAT3, an oncogenic pathway that endows CLL cells’ survival advantages by binding and activating the promotors of LPL (145, 146). Additionally, further study has revealed that LPL in aggressive CLL cells generates activating ligands for the nuclear receptor PPARα and allows fatty acids to be employed as fuel (147). Thus, STAT3-mediated aberrant LPL in CLL can serve as an ideal targeted marker for blocking lipid metabolism.

## Impact of Metabolic Regulation on Anti-Leukemia Immune Responses

**Figure 2:**
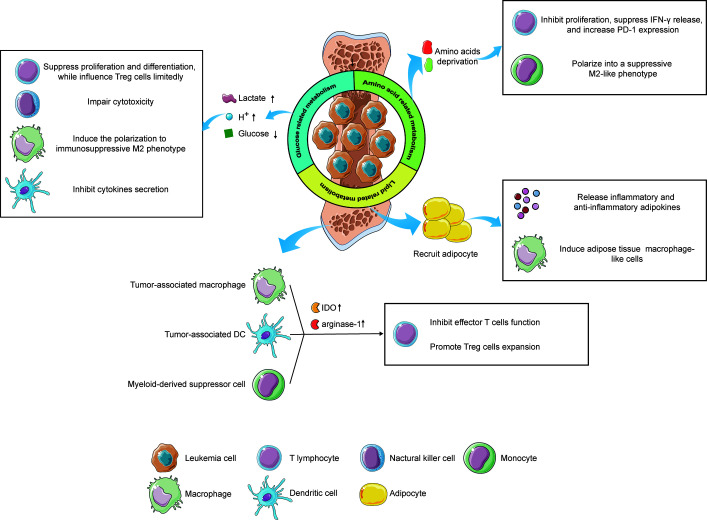
The influence of metabolic alterations of leukemia cells and immune cells on the immune responses under the microenvironment. Reprogramming glucose-related metabolism impacts immunity *via* releasing lactate and hydrogen ions, which create an acidic microenvironment, as well as depriving of extracellular glucose, resulting in an immunosuppressive status of filtrating immune cells. Furthermore, leukemia cells also destitute the amino acids in the microenvironment, leading to the inactivation of T lymphocytes and phenotype shift of monocytes. Lastly, by recruiting adipocytes in the BMM *via* the lipid-related pathway, leukemia cells reveal a potential effect on immunological regulation. Immune cells, like tumor-associated macrophages, tumor-associated DCs, and MDSCs can also impact the T cells’ function *via* upregulating the expression of IDO and arginine-1, contributing to an immune-tolerant microenvironment. The figure was produced with the assistance of Servier Medical Art (https://smart.servier.com).

Several research have revealed that altered cellular metabolism not only changes the phenotype of leukemia cells but also impacts the BMM greatly, resulting in the initiation, progression, and metastasis of leukemia (148). As a dynamic and complex biological tissue, the bone marrow niche influences the anti-leukemia immune responses significantly *via* cellular or noncellular components in the tumor microenvironment (TME) (**Figure 2**). For instance, by depriving essential nutrients from the bone marrow niche, leukemia cells fabricate a long-term nutrients deficiency microenvironment, resulting in the dysfunction of immunocytes, leading to a suppression of antileukemia immune responses. Although the current understanding of the relationship between metabolic regulation in the leukemia-resided BMM and antileukemia immunity is still incomplete, a deep investigation of this field will undoubtedly open a therapeutic avenue for leukemia immunotherapy.

As the preferential glucose metabolism of leukemia, aerobic glycolysis generates and secretes a high level of extracellular lactate and hydrogen ions, these products in turn, acidify the microenvironment. The acidic environment is beneficial to the invasion of leukemia cells, while detrimental to immune cells that target leukemia cells (149). Previous studies exhibited that a high level of extracellular lactate suppresses the differentiation from monocytes to dendritic cells (DC) and inhibits the cytokine secretion of DCs and effector T cells (150, 151). Furthermore, elevated lactate in the microenvironment attenuates the secretion of lactate from T cells because the transportation of lactate depends on the difference between intracellular and extracellular concentration in T cells, leading to the accumulation of intracellular lactate in T cells, which inhibits the cytotoxic T cell function (152). Additionally, an acidic microenvironment induces the polarization of macrophages from M1 to immunosuppressive M2 phenotype, which supports leukemia angiogenesis and metastasis (149). Meanwhile, a low-pH microenvironment also impairs the cytotoxicity of NK cells. Huge consumption of glucose by leukemia cells causes glucose deficiency in the microenvironment under limited blood supplement, inhibiting the proliferation and differentiation of tumor-infiltrating lymphocytes *via* glucose competence (153). However, glucose competition has limited effects on regulatory T (Treg) cells due to the predominant role of lipids metabolism in the energy production of Treg cells. Thus, under glucose-deprived conditions, Treg cells still exert consistent immunosuppressive functions while effector T cells show a relatively weakened antileukemia capability, which is favorable for the progression of leukemia (154). Taken together, neutralizing the acidic BMM and suppressing glucose deprivation of leukemia cells can serve as novel strategies against leukemias, prompting immune surveillance and clearance.

Amino acids provide building blocks for the synthesis of proteins and other compoundsnecessary for structure construction, signaling transduction, and etc. Deprivation of amino acids in the BMM by leukemia cells profoundly impacts the immune cells, leading to an immunosuppressive microenvironment eventually. For instance, IDO was found to be highly expressed and abnormally activated in leukemias, exhibiting its inhibitory effects on T cells *via* tryptophan deprivation (155). Moreover, leukemia cells can induce immune tolerance by increasing the expansion of Treg cells in an IDO-dependent manner (155, 156). In addition to leukemia cells, mesenchymal stem cells (MSCs), critical contributors to tissue inflammation homeostasis, were evidenced to utilize tryptophan-depleting IDO to suppress T cells *via* STAT1 glycosylation. Interfering with STAT1 activity in MSCs impacts IDO upregulation, further impeding T cell suppression (157). Arginine, a non-essential amino acid, also participates in the regulation of immune responses. Enhanced arginine metabolism in AML blasts contributes to the secretion of arginase II in the microenvironment and a high concentration of arginase II in the plasma has been evidenced in patients with AML (158). The low arginine microenvironment created by AML blasts acts as an immune brake resulting in T cells anergy, including impaired proliferation, reduced IFN-γ release, and up-regulation of PD-1 (159). Furthermore, AML blasts show an arginase-mediated ability to polarize monocytes into a suppressive M2-like phenotype and hamper the proliferation and differentiation of human CD34^+^ progenitors (158). Another key amino acid, glutamine, was also reported to be involved in immunological regulation. Depletion of glutamine impedes proliferation and cytokine production of T cells and this cannot be salvaged by offering biosynthetic precursors of glutamine (160). Paradoxically, a recent study revealed that tumor-infiltrating CD8^+^ T cells, under glutamine-restricted conditions, exhibit a boost in proliferation with an increased expression of Ki67, whereas T cells exhaustion was limited (161). Therefore, an integrated understanding of the impacts of glutamine metabolism on the BMM, even on immunological regulation towards leukemias, is urgently needed to clarify the underlying mechanism by which glutamine influences immune responses.

The BMM is an area filled with numerous adipocytes. When tumor cells migrating from other tissues are located in the BMM, adipocytes will be recruited and deliver lipids to tumor cells *via* the CD36 receptor (162). In addition, these adipocytes secrete a set of inflammatory adipokines, such as leptin, tumor necrosis factor-alpha (TNF-α), and IL-6, as well as an anti-inflammatory adipokine, called adiponectin, to modulate the immunological microenvironment (163). Furthermore, adipocytes have been found to induce the differentiation of bone marrow precursors into adipose tissue macrophage-like cells *via* releasing exosome-sized, lipid-filled vesicles (164). These results implicit lipids metabolism regulated by adipocytes might be involved in antileukemia immune responses. Besides, the existence of lipid intermediates in ascites of ovarian cancer patients induced a proliferation arrest of ascites-derived lymphocytes in the G0/G1 phase, implying that targeting lipid metabolism in the microenvironment can serve as a therapeutic option to struggle against tumors *via* regulation of immune responses (165). However, our current understanding concerning the relationship between lipid metabolism and antileukemia immunity remains elusive and in-depth investigations correlated with this field are encouraged to broaden the knowledge.

Nevertheless, not only does the reprogramming metabolic wiring of leukemia cells influences the leukemic niche but also the alteration of metabolism in some immune cells impacts the BMM, contributing to an immunosuppressive microenvironment that favors the progression of leukemia (**Figure 2**). As we mentioned above, acidification polarizes infiltrating macrophages into the tumor-supporting M2 phenotype, also known as “ tumor-associated macrophages” (TAMs). TAMs exhibit atendency of upregulation of transcription factor HIF1α, which in turn enhances the expression of vascular endothelial growth factor (VEGF) and arginase-1, underlining the tumor-favoring function of TAMs (166). Furthermore, an elevated IDO expression has been evidenced in TAMs (167). TAMs induce the proliferation of Treg cells *via* tryptophan catabolite, leading to the suppression of T cells function and immune-tolerant microenvironment (168). Another immunosuppressive cell phenotype, tumor-associated DCs (TADCs), has also been reported to participate in the modulation of the microenvironment *via* metabolic alterations. By enhancing the expression of arginase-1 and IDO, which delete the arginine and tryptophan in the TME, TADCs attenuate the tumor-fighting function of effector T lymphocytes, supporting the immune evasion of leukemia cells (169). The role of myeloid-derived suppressor cells (MDSCs) in the regulation of the TME *via* metabolic rewiring also could not be ignored. It has been reported that DMSCs overexpress IDO to catalyze tryptophan into kynurenine, suppressing T cells function and promoting Treg cells generation (170). These facts together imply that the metabolic reprogramming of immune cells also play a pivotal role in the modulation of the BMM, highlighting the promising possibility to target immune cells metabolism for leukemia treatment.

## Exploiting of Metabolism Targeting Treatments in Leukemia

As discussed above, altered metabolism is essential in hematologic malignancies to overcome survival stress and adjust to the diverse microenvironment. Thus, therapy against leukemias by targeting reprogramming metabolic molecules seems to be an ideal option to struggle against hematological malignancies and the selection of specific biomarkers is critical in the development of targeted therapeutic agents (171). Recently, researchers are dedicated to identifying novel metabolism-targeting drugs. Metabolic enzymes and the components within the glucose, amino acid, and fatty acid metabolic pathways, are regarded as attractive targets (172), and some of them have shown inspiring results in clinical trials, as shown in **Table 1**.

**Table 1:** Metabolism-targeted agents and their antileukemia effects *in vitro* and *in vivo*.

Substance	Phase	Classification of leukemia	Targeted biomarker	Combination	Study results	Reference
Ritonavir	Preclinical	CLL	GLUT4	Metformin	Ritonavir inhibited the expression of GLUT4 and significantly reduced glucose transportation, oxygen consumption, therefore inducing CLL cells apoptosis.	(173)
2-DG	Preclinical	AML	HK2	Sorafenib	2-DG caused apoptotic AML cell death in a dose-dependent manner. Moreover, 2-DG plus sorafenib provided a significant therapeutic benefit over sorafenib alone.	(174)
6-AN	Preclinical	AML	G6PD	cytarabine	6-AN effectively induced apoptosis of AML cells and inhibited tumor proliferation *in vivo* without apparent systemic toxicity. 6-AN and cytarabine synergized to induce cytotoxicity against AML cells.	(175)
DMAMCL	Preclinical	AML	PKM2	None	DMAMCL effectively promoted PKM2 tetramer formation and prevented nucleus translocation, impairing AML cells proliferation and migration *in vivo*.	(176)
Oxamate	Preclinical	ALL	LDHA	None	Oxamate targeted LDHA, thereby inhibiting ALL progression through the c-Myc-ROS and PI3K/AKT/GSK3β signaling pathways	(177)
AR-C155858, Syrosingopine	Preclinical	AML	MCT1, MCT4	Arabinosylcytosine	AR-C155858 and syrosingopine inhibited lactate metabolism and leukemic cell proliferation *via* inhibition of MCT1 and MCT4, and enhanced cytotoxicity of arabinosylcytosine in AML cells	(178)
GPNA	Preclinical	AML	ASCT2	None	GPNA effectively suppressed leukemia progression and greatly increased apoptosis in leukemic cells in both peripheral blood and bone marrow of AML xenograft mice	(128)
CB-839	I	AML, ALL	GLS	None	One patient achieved a CR and has been on study for more than 10 months; Four additional patients remained on study for at least 12 weeks. Three out of 18 patients experienced grade 3 AEs.	(179)
						NCT02071927
PEG-asparaginase	Approved for ALL	ALL	Asparagine	Chemotherapy agents	Four-year continuous CR for leukemia patients was 68% with asparaginase treatment as compared to 55% without asparaginase treatment and toxicities were tolerable.	(180)
ADI-PEG20	II	AML	Arginine	None	PFS and OS were 1.8 months and 7.9 months in AML patients after receiving ADI-PEG20. Among 21 evaluable patients, two achieved CR while seven achieved SD.	(181)
						NCT01910012
ADI-PEG20	I	AML	Arginine	Cytarabine	The median OS in 18 AML patients was 8.0 months, with an ORR of 44.4%. The ORR reached 71.4% and the CR rate reached 57.1% in seven treatment- naïve patients.	(182)
						NCT02875093
Indoximod	I	AML	IDO	Cytarabine	Among 25 AML patients, 21achieved remission. Among 12 patients with MRD available in remission, 10 had MRD negative status. Indoximod combined with cytarabine was well-tolerated.	(183)
						NCT02835729
AIC-47	Preclinical	CML, ALL	CPT1	Imatinib	AIC-47 inhibited fatty-acid metabolism *via* suppression of CPT1, while AIC-47 and imatinib in combination exhibited a significant synergic cytotoxicity toward leukemia cells.	(184)
Etomoxir	Preclinical	AML	CPT1	ABT-737	Etomoxir limited the proliferation of AML cells and sensitized leukemia cells to apoptosis induction by ABT-737 *in vitro*. *In vivo*, combination therapy effectively decreased tumor burden and prolonged median survival.	(185)
Orlistat	Preclinical	CLL	Lipase	fludarabine	Lipase inhibitor orlistat induced apoptosis and impeded the proliferation of CLL cells. Simultaneous incubation with fludarabine enhanced the cytotoxic effects of orlistat.	(186)
Enasidenib	Approved for AML	AML	mutant IDH2	None	Among patients with relapsed or refractory AML, the ORR was 40.3%. Median OS was 9.3 months in 176 patients and 19.7 months in 34 patients with CR. In 130 patients with IDH2-R140 mutations, ORR was 35.4% while ORR was 53.3% in 45 patients with IDH2-R172 mutations.	(187)
						NCT01915498
Ivosidenib	Approved for AML	AML	mutant IDH1	None	The CR plus CRh rate was 42.4% in 34 patients newly diagnosed with AML. Median OS was 12.6 months while the estimated 12-month OS rate was 51.1%. Ivosidenib monotherapy was well-tolerated.	(188)
						NCT02074839

2-DG, 2-Deoxy-d-Glucose; 6-AN, 6-aminonicotinamide; DMAMCL, dimethylaminomicheliolide; GPNA, L-c-glutamyl-p-nitroanilide; PEG-asparaginase, pegylated asparaginase; GLUT 4, glucose transporter 4; HK2, hexokinase-2; G6PD, glucose-6-phosphate dehydrogenase; PKM2, pyruvate kinase isozymes M2; LDH, lactate dehydrogenase; MCT, monocarboxylate transporters; ASCT2, glutamine transporter encoded by Solute Carrier Family 1 Member 5 gene; GLS, glutaminase; IDO, Indoleamine-pyrrole 2,3-dioxygenase; CPT1, carnitine palmitoyltransferase I; IDH, isocitrate dehydrogenase; CR, Complete remission; CRh, CR with partial hematologic recovery; OS, overall survival; PFS, Progression-free survival; SD, Stable disease; CCR, Complete remission rate; ORR, Overall response rate; MRD, Measurable residual disease; AE, Adverse event.

### Targeting Glycolysis

Generally, most of the glucose consumed by leukemia cells is catabolized through glycolysis. Thus, preventing glucose uptake and glycolysis will be an effective strategy to struggle against leukemia. GLUT mediates the glucose uptake and transportation of leukemia cells. By inhibiting GLUT4, protease inhibitor ritonavir exhibits similar toxic effects on CLL cells as glucose deprivation does *in vitro* (173). Furthermore, when co-incubating with metformin, an inhibitor of mitochondrial complex I activity, ritonavir-resistant CLL cells are sensitized, resulting in the elimination of CLL cells *via* perturbation of glucose metabolism. Promising antileukemia results have also been evidenced by targeting rate-limiting enzymes related to glycolysis and the PPP, such as HK2 (174), G6PD (175), PKM2 (176). Leukemia cells prefer shunting pyruvate into lactate production to adjust to rapid energy consumption rather than into the TCA cycle for further OXPHOS, providing a probability to target LDH to inhibit the production of lactate in leukemia cells. The LDHA inhibitor oxamate fulfills its antileukemia effects by arresting ALL cells in G0/G1 phase and inducing apoptosis (177). Further mechanism exploration revealed that blocking LDHA also leads to the inactivation of the c-Myc and PI3K signaling pathway, suggesting that targeting LDH is an ideal selection to achieve antitumor benefits when applying *in vitro*. Last but not least, prohibiting monocarboxylate transporters (MCTs), the transporter of lactate, will results in the accumulation of glycolytic end-products including lactate and H^+^, which consequently, lead to intracellular acidification and apoptosis. A recent study demonstrated that AR-C155858 and syrosingopine, inhibitors of MCT1 and MCT4, exert an anti-proliferative effect and enhance AML cells’ sensitivity to chemotherapeutic agents (178). Altogether, the results highlight the important role of targeting the glycolysis pathway in the treatment of leukemia. Regretfully, clinical results concerning glycolysis-related inhibitors to cure leukemia are rare up to now so far. The bio-safe and efficacy of glycolysis-related inhibitors for leukemia treatment are eager to explore clinically.

### Targeting Amino Acid Metabolism

The current development of targeted agents against glutamine metabolism of cancer cells focuses on membrane glutamine transporter inhibition, GLS inhibition, and glutamine depletion. ASCT2 mediates the entry of glutamine and other amino acids into cells. ASCT2 inhibitor GPNA showed potential in decreasing survival and promoting apoptosis of AML cells *in vitro*. In AML mice, GPNA treatment effectively suppressed leukemia progression. Attenuated splenomegaly and hepatomegaly, and reduced AML cells infiltration were observed. Notably, the impact of GPNA on normal blood cell formation was limited (128). As a selective and irreversible inhibitor of GLS, CB-839 has shown ideal antileukemia effects on AML and ALL cells *in vivo* (129). Furthermore, a completed phase I clinical trial also evidenced the potential role of CB-839 to combat leukemias (NCT02071927) (179). Eighteen patients, including AML (n=16), ALL (n=1) and mixed leukemia (n=1), were enrolled in this trial. Results showed that CB-839 was well tolerated in advanced leukemia patients. One AML patient achieved a complete remission (CR) and has been on study for more than 10 months, while four additional patients remained on study for at least 12 weeks. Another Phase Ib/II clinical trial has demonstrated that CB-839 is safe and effective in combination with azacitidine in patients with advanced myelodysplastic syndrome (MDS) (NCT03047993) (91). Among ten patients with MDS, seven patients achieved CR, two patients had stable disease (SD), while one patient had no response, suggesting that GLS inhibition may induce a robust effect against hematopoietic malignancies synergetically when combining with other conventional treatments.

Asparaginase has been approved by FDA as the frontline therapeutic agent used in ALL. A previous clinical trial revealed that 357 patients with ALL reached a 68% of four-year continuous CR rate after receiving asparaginase treatment, while the rate was only 55% in patients without asparaginase (180). The overall one-sided logrank test favors the asparaginase group over the control in ALL treatment. Toxicities related to asparaginase were manageable.

Many types of cancer cells, including AML disease, depend on the exogenous supply of arginine due to the defects in arginine synthetase (189, 190). Therefore, arginine depletion is a possible therapeutic option for leukemia cure. A phase II clinical trial reported that two patients reached CR while seven patients achieved SD among 21 argininosuccinate synthetase (ASS)-deficient AML patients after receiving arginine deiminase (ADI-PEG20) (NCT01910012) (181). The median progression-free survival (PFS) and overall survival (OS) were 1.8 months and 7.9 months, respectively. Clinical outcomes and further transcriptome sequencing implied that ADI-PEG20 monotherapy is required but not sufficient for ASS-deficient leukemia treatment. A rational combination with other targeted or conventional drugs may improve the efficacy of the antileukemia response as a whole. Recently, another phase I study combining ADI-PEG20 and low-dose cytarabine for the treatment of AML has completed and encouraging outcomes have been observed (NCT02875093) (182). Results revealed that the overall response rate (ORR) in 18 patients was 44.4%, with a median OS of 8.0 months. In seven treatment-naive patients, the ORR was 71.4% and the CR rate was 57.1%. Moreover, patients suffered limited fatal side effects after receiving the combination of ADI-PEG20 and cytarabine. Taken together, ADI-PEG20 shows the superior clinical performance when cooperating with conventional clinical agents for AML therapy, and the underlying mechanism of combination warrants further explorations.

Increased IDO activity results in T cells inactivation and immunosuppression (191). Even though IDO inhibitors are mainly being tested in solid tumors, clinical trials have also started to test this potential biomarker in AML (NCT02835729) (183). Among 25 AML patients who received more than one dose of indoximod, a small-molecule inhibitor of the IDO pathway, 21 AML patients attained disease remission. Furthermore, ten patients experience measurable residual disease (MRD) negative among twelve patients with MRD available in remission. When combining with standard AML induction therapy, indoximod is well tolerated. These promising results indicated that indoximod could serve as an ideal targeted agent to combat leukemia by inhibiting IDO activity.

### Targeting Fatty Acid Metabolism

To meet the exquisite demands of cell growth and proliferation, leukemia cells exhibit high dependence on FA metabolism, making it possible to target this metabolic vulnerability to eliminate leukemia cells (192). Currently, FAO inhibitors are being tested in preclinical trials. Imatinib induces the inactivation of BCR-ABL, thereby suppressing glycolysis in CML cells. However, compensatory FAO activation rescued glucose-independent CML cells’ survival after imatinib treatment. AIC-47 suppressed the expression of CPT 1 and directly fatty acid metabolism, contributing to the complementary attack on CML energy metabolism when combining with imatinib (184). Moreover, another CPT 1 inhibitor, etomoxir, has been reported to exhibit significant antiproliferative effects on AML cells and sensitize AML cells to apoptosis induced by ABT-737 *in vitro* (185). Although FAO inhibitors have not been practiced clinically, preclinical studies already reveal the feasibility of appliance of FAO inhibitors in leukemia treatment.

In CLL, LPL has been identified as a specific biomarker, which indicates an unfavorable prognosis in patients (193, 194). *Via* targeting LPL molecule, orlistat induced apoptosis of CLL cells, while limited cytotoxicity was demonstrated in healthy B cells and peripheral blood mononuclear cells (186). Moreover, the antileukemia effects of orlistat on CLL cells strengthened when incubated with fludarabine, simultaneously.

### Targeting Isocitrate Dehydrogenase Mutation

Retrospective studies claim that the IDH mutation was associated specifically with a poorer prognosis in AML (195–197). Currently, ivosidenib and enasidenib, inhibitors of IDH1 and IDH2, have become the first FDA-approved metabolism targeting drugs for AML. In a phase I/II trial, the efficacy of enasidenib was examined in patients with mutant-IDH2 advanced myeloid malignancies (187). The median overall survival among patients with relapsed/refractory AML was 9.3 months while patients who reached CR or partial remission had a median survival of 19.7 months or 14.4 months, respectively. The ORR for all relapsed/refractory AML patients was 40.3%, with a median response duration of 5.8 months. This study proved that daily enasidenib therapy induces antileukemia responses and is well-tolerated in patients who failed in conventional treatment previously. Ivosidenib, a mutant IDH1 specific inhibitor, has been evaluated in a phase I trial (NCT02074839) (188). Thirty four patients with AML were enrolled in this trial and their median overall survival was 12.6 months with a median follow-up period of 23.5 months. CR plus CR with partial hematologic recovery rate was 42.4%. The most commonly reported therapy-related serious adverse event was IDH differentiation syndrome. These results demonstrated that enasidenib and ivosidenib induce durable remissions and are safe in mutant IDH AML treatment.

## Conclusion and Prospectives

In this review, we summarized the alteration of metabolic events supplementing building blocks and energy for rapid proliferation, drug resistance, self-renewal, etc. during leukemogenesis. Deep explorations into leukemia metabolomics have extended a novel therapeutic path through which targeting leukemia metabolic vulnerabilities exerts prominent antileukemia effects. Furthermore, the importance of cross-talk between leukemia cells and immunocytes has been highlighted in the text, especially under complicated BMM conditions. Since metabolism participates in immunological regulation and usually results in an immunosuppressive microenvironment favoring the occurrence and development of leukemia cells, metabolic interventions could serve as a potential option to improve immune response toward leukemia, particularly when combined with immune regulatory agents. Although clinical benefits were evidenced in patients with leukemia by targeting metabolic molecules, the effectiveness is still far from satisfying and cytotoxic side effects are still unignorable. Thus, exploring leukemia-specific metabolic dependencies to identify potential biomarkers with limited toxicities toward normal cells will be the future research focus. Moreover, different subsets of leukemia cells harbor individual genetic and biological features, therefore exhibiting different metabolism mechanisms, which means targeting metabolic vulnerabilities should be subgroup-specific, even individual-specific, to achieve the greatest effect. The application of accurate metabolic targeting requires sophisticated new techniques to elucidate the complexity and dynamicity of remodeling of metabolic wiring. Additionally, LSCs are hypothesized to be responsible for the conventional treatment resistance and leukemia relapse. Compared with normal leukemia cells, LSCs reveal a different metabolic preference, emphasizing that a comprehensive understanding of the metabolic status of LSCs will also provide us novel insights into therapy against drug-resistant or relapsed leukemia. Last but not least, a rational combination therapeutic strategy, for instance, integrating targeted agents related to metabolic pathways and conventional chemotherapies, is worth further exploration clinically and may improve the cure rate of leukemia comprehensively.

## Author Contributions

MGH and AB contributed to conception and design of the study. MS, YZ, and ZX wrote sections of the manuscript. All authors contributed to the article and approved the submitted version.

## Conflict of Interest

The authors declare that the research was conducted in the absence of any commercial or financial relationships that could be construed as a potential conflict of interest.

## Publisher’s Note

All claims expressed in this article are solely those of the authors and do not necessarily represent those of their affiliated organizations, or those of the publisher, the editors and the reviewers. Any product that may be evaluated in this article, or claim that may be made by its manufacturer, is not guaranteed or endorsed by the publisher.
